# A phase II trial of concurrent chemoradiotherapy with weekly paclitaxel and carboplatin in advanced oesophageal carcinoma

**DOI:** 10.1007/s10147-018-1240-4

**Published:** 2018-02-12

**Authors:** Yi Xia, Yun-hai Li, Yun Chen, Qi Liu, Jun-hua Zhang, Jia-ying Deng, Ta-shan Ai, Han-ting Zhu, Harun Badakhshi, Kuai-Le Zhao

**Affiliations:** 1 Department of Radiation Oncology, Fudan University, Shanghai Cancer Center, 270 Dong’An Road, Shanghai, 200032 China; 2 Department of Radiation Oncology, Fudan University, Shanghai Cancer Center Minhang Branch, 106 RuiLi Road, Shanghai, 200240 China; 3 Department of Clinical Radiation Oncology, Ernst von Bergmann Medical Center, D-14467 Potsdam, Germany

**Keywords:** Oesophageal carcinoma, Paclitaxel, Carboplatin, Chemoradiotherapy

## Abstract

**Background:**

This study was performed to assess the efficacy and feasibility of definitive chemoradiotherapy consisting of weekly doses of combined paclitaxel and carboplatin concurrent with radiation therapy, followed by 2 cycles of consolidation chemotherapy for advanced esophageal carcinoma.

**Methods:**

Eligibility criteria included local, advanced, newly diagnosed and postoperative local regional lymph node metastasis; Eastern Cooperative Oncology Group (ECOG) score ≤ 2; and adequate organ function. Patients received concurrent chemoradiation therapy consisting of radiotherapy (50.4 Gy/28 Fx or 61.2 Gy/34 Fx) and concurrent paclitaxel (50 mg/m^2^) and carboplatin (area under the curve, AUC = 2) on days 1, 8, 15, 22 and 29. The two-cycle consolidation chemotherapy protocol was paclitaxel (175 mg/m^2^) plus carboplatin (AUC = 5) administered on days 57 and 85, after concurrent chemoradiotherapy.

**Results:**

Between August 2013 and February 2015, 65 patients with oesophageal carcinoma were enrolled in the study; 34 (52.3%) were newly diagnosed and 31 (47.6%) had postoperative local regional lymph node metastasis. The median overall survival time was 21.7 months (95% confidence interval [CI] 16.7–26.6), and the median progression-free survival time was 12.1 months (95% CI 9.0–15.3). A total of 96.9% (63/65) and 67.6% (44/65) patients completed ≥5 cycles and all 7 cycles of chemotherapy, respectively. A total of 93.8% (61/65) patients completed radiation therapy. The 1- and 2-year overall survival rates were 73.7 and 42.0%, respectively. The 1- and 2-year progression-free survival rates were 50.6 and 31.1%, respectively. Grade 3–4 toxicity during chemoradiotherapy included neutropenia (24.5%), thrombocytopenia (4.6%), fatigue (1.5%), anaemia (1.5%), radiation dermatitis (1.5%), pneumonitis (1.5%), oesophagitis (4.6%) and vomiting (1.5%).

**Conclusions:**

In patients with locally advanced oesophageal cancer, the combination of weekly doses of paclitaxel and carboplatin was well tolerated and produced comparable results. A three-arm randomised phase III trial (NCT02459457) comparing paclitaxel in combination with cisplatin, carboplatin or 5-fluorouracil with concurrent radiotherapy is on-going at our hospital.

## Introduction

Oesophageal cancer is highly malignant with a propensity for high rates of local recurrence and metastasis. Although significant advances in staging and treatment of patients have recently been seen, this malignancy is still lethal for the majority of patients [1]. Definitive concurrent chemoradiotherapy is the standard treatment for non-surgery patients with oesophageal cancer, and the use of cisplatin with 5-fluorouracil (5-FU) is the most common chemotherapy regimen [2]. However, after definitive chemoradiation therapy, 5-year survival rates are poor (26%) [3]. In addition, 20% of patients who received chemoradiation experienced life-threatening side effects, and chemotherapy could not be completed in >40% of the patients [4]. Thus, more sensitive, less toxic chemotherapy regimens are urgently needed.

Paclitaxel is a promising agent in oesophageal cancer, with response rates of approximately 32% with single-drug treatment in locally advanced and metastatic patients [5]. Paclitaxel is a mitotic inhibitor that blocks cells in the G2M phase of the cell cycle, the most radiosensitive phase, with a sensitising enhancement ratio of 1.48 [6]. The investigation of a paclitaxel-based regimen and administration route is important for improving survival and reducing side effects. Effective and well-tolerated chemotherapy can prolong overall survival and improve quality of life.

The combination of paclitaxel and carboplatin (TC) with concurrent radiotherapy has been tested in patients with advanced non-small-cell lung cancer [7–9]. In several of these studies, the combination of paclitaxel and carboplatin was given weekly with concurrent radiotherapy, followed by two or four cycles of consolidation chemotherapy. The overall response rate varied from 71 and 79%. The major toxicity was oesophagitis: in 10–46% of the patients a grade 3 or 4 oesophagitis was found. Treatment with paclitaxel and carboplatin and concurrent radiotherapy can be given on an outpatient basis, which is advantageous. Furthermore, this regimen is probably less toxic than cisplatin-based therapy. Van Meerten et al. reported a phase 2 study of neoadjuvant chemoradiotherapy (nCRT) consisting of weekly administration of carboplatin and paclitaxel with concurrent radiotherapy [10]. The regimen was associated with a low rate of serious toxic effects, and a complete resection with no tumor within 1 mm of the resection margins (R0) was achieved in all patients who underwent resection. In the CROSS study, which included 366 patients with esophageal and gastro-oesophageal junctional tumors, the complete resection rate was increased from 69% with surgery alone to 92% with the combined therapy, with no increase in the preoperative mortality rate. Ultimately, the nCRT in this trial’s population provided a highly significant 34% reduction of the risk of death, as well as a significant 42% reduction of the risk of relapse [11].

In comparison with cisplatin, carboplatin has a lower incidence of neurotoxicity, and is easier to administer in outpatients. Pharmacokinetic studies of paclitaxel administered with carboplatin demonstrate that area under the curve (AUC)-guided dosing can be accurately predicted by the Calvert formula. On the basis of these data, we began a phase II feasibility study to evaluate the overall survival (OS), progression-free survival (PFS) and toxicities in advanced oesophageal cancer patients treated with weekly paclitaxel and carboplatin and concurrent radiotherapy.

## Patients and methods

### Patient selection

The eligibility criteria for the study were as follows: (1) age ≤ 75 years; (2) Eastern Cooperative Oncology Group (ECOG) score 0–2; (3) cytologically or histologically confirmed oesophageal cancer; and (4) locally advanced, newly diagnosed patients with T2-4NxM0-1a or TxNxM1b (supraclavicular lymph node metastases for middle and lower thoracic oesophageal cancer or mediastinal lymph node metastases for cervical oesophageal cancer; without organ metastases according to the 6th edition of UICC) or postoperative local regional lymph node metastasis patients who did not receive chemotherapy or radiotherapy.

Patients were required to meet the following laboratory criteria: adequate bone marrow function (neutrophil count > 2.0 × 10^9^/L, white blood cell count > 4.0 × 10^9^/L and platelet count > 100 × 10^9^/L), normal renal function and normal liver function. Patients with tracheoesophageal fistula, organ metastasis or complete obstruction were not eligible for this study. All patients underwent complete examination and nutritional assessment. The disease evaluation included upper endoscopy, chest and abdominal computed tomography (CT), ultrasonography and barium oesophagram examination to eliminate distant organ metastasis.

### Ethical considerations

Written informed consent was obtained from all patients before pre-study assessments, and the study protocol was approved by the Ethics Committee of the Cancer Hospital Affiliated to Fudan University.

### Treatment schedule

**Fig. 1:**
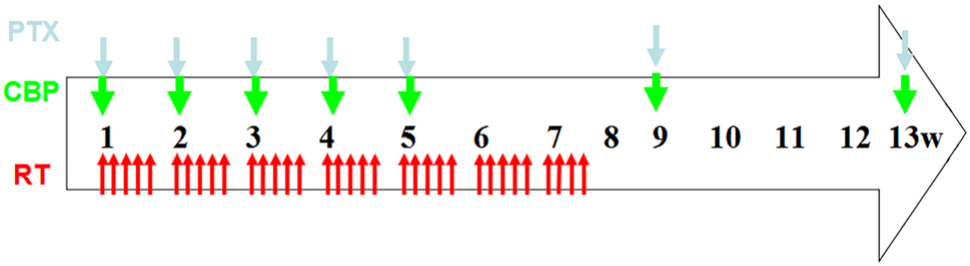
Treatment schedule. Radiation therapy (RT): total dose of 61.2 Gy/34 Fx, 1.8 Gy/Fx, 5 days/week. Concurrent chemotherapy: paclitaxel (PTX) 50 mg/m^2^ and carboplatin (CBP) AUC = 2 on days 1, 8, 15, 22 and 29. Consolidation chemotherapy: PTX 175 mg/m^2^ plus carboplatin AUC = 5 was given on week 9 and 13

The chemotherapy regimen consisted of a combination of paclitaxel and carboplatin. Concurrent chemotherapy was administered from the first day of radiotherapy and comprised five cycles of paclitaxel (50 mg/m^2^) in a 3-hour infusion and infused carboplatin (AUC = 2) on days 1, 8, 15, 22 and 29. Thirty minutes before treatment with paclitaxel, patients were pre-medicated with 10 mg of dexamethasone and 300 mg of cimetidine intravenously and 25 mg of promethazine intramuscularly. The two-cycle consolidation chemotherapy protocol was paclitaxel (175 mg/m^2^) plus carboplatin (AUC = 2) on weeks 9 and 13. Pre-treatment before paclitaxel treatment was as follows: 25-mg promethazine intramuscular injection at 0.5 h, 27 tablets of dexamethasone administered orally (0.75 mg/tablet) at 12 and 6 h before treatment, and 300 mg of cimetidine administered intravenously at 0.5 h before paclitaxel treatment. If grade ≥3 haematological toxicity occurred and persisted, chemotherapy was suspended until recovery, and the regimen dose was sequentially reduced by 25%. The treatment modality is illustrated in Fig. 1.

Radiation therapy was administered using intensity-modulated radiation therapy (IMRT). Planning was performed using a Megavoltage simulator with a photon energy of 6 MV. For previously untreated diagnosed patients and recurrent patients, the gross tumour volume (GTV) was defined as the volume of the primary tumour observed on oesophageal barium exam, upper digestive endoscopy and CT. Metastatic lymph nodes were defined as lymph nodes ≥1 cm in the shortest axis and ≥5 mm in the tracheo-oesophageal groove. The clinical target volume (CTV) was defined by adding 3-cm margins of proximally and distally uninvolved oesophagus without including lateral margins and lymph nodes. The planning target volume (PTV) was calculated by adding 1-cm margins around the CTV, but the margins were reduced when the PTV covered the spinal cord. All patients were treated with a total dose of either 61.2 Gy in 34 fractions or 50.4 Gy in 28 fractions. The lower dose was used when patients had abdominal lymph node metastasis or tumour in excess of the limitations of normal organs, such as the lungs, spiral cord and intestines.

Plan optimisation was as follows: (1) 99% of the PTV was covered by 95% of the prescribed dose; (2) 95% of the PTV volume was covered by the prescribed dose; (3) the maximum dose did not exceed 110% of the prescribed dose in a continuous volume of <1 cm^3^ in the PTV; and (4) the maximum dose of the PTV did not exceed 110% of the prescribed dose in a continuous volume of <1 cm^3^. The normal tissue constraints of the critical organs were as follows: a maximum spinal cord point dose of ≤45 Gy; lung V20 (percentage of total lung volume receiving ≥20 Gy) of ≤30% and mean lung of ≤16 Gy; mean heart dose of ≤30 Gy; and maximum intestine dose of ≤50 Gy.

### Follow-up

In the first and second year after treatment completion, the patients were seen at the outpatient clinic every 3 months for a history and clinical examination. During the third year after treatment, follow-up occurred every 6 months until year 5. If applicable, disease recurrence, late toxic effects or death was documented. Adverse reactions to chemoradiotherapy were evaluated according to the National Cancer Institute Common Toxicity Criteria version 4.0 (NCI-CTC 4.0).

### Statistical methods

The primary end point of this study was OS. Secondary end points comprised assessment of safety and compliance, PFS and local control (LC). Local failure was defined as recurrence or persistence within the radiation therapy PTV. Recurrence outside the treatment volume was defined as distant. OS was calculated from the first day of chemoradiation therapy to the time of the last follow-up or death. PFS was defined as the time from chemoradiotherapy day 1 to progression, death or last follow-up. We hypothesised that the addition of paclitaxel and carboplatin would increase the 1-year overall survival rate. A sample size of 59 was required to detect an increase in the 1-year survival rate from 57 to 73% with a power of 80% and α error (two-sided test) of 0.05. A 10% adjustment for drop-outs resulted in a sample size of 65 patients. Data were analysed according to the intention-to-treat population. Kaplan–Meier curves were fitted to estimate the OS and failure-free local control rates. Median survival estimates were calculated. Statistical analyses were performed using SPSS 20.0.

## Results

### Patient characteristics

**Fig. 2:**
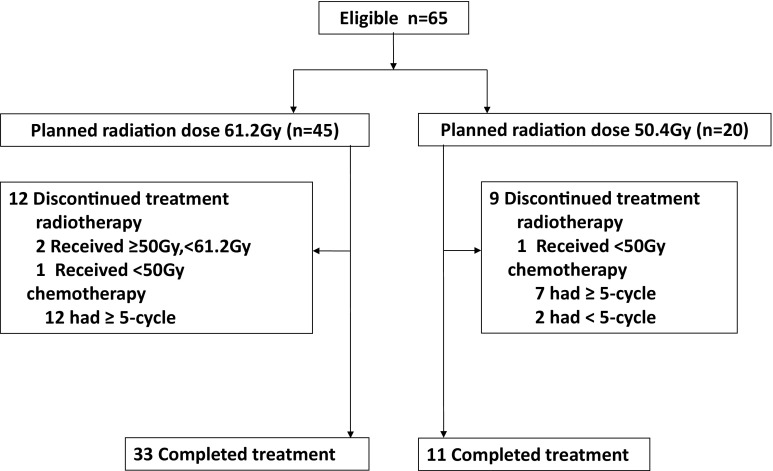
CONSORT diagram of patient progress through the study

Between August 2013 and February 2015, 65 patients were enrolled in our study. Figure 2 shows the progression through the study phases. The baseline characteristics of the 65 patients are shown in Table 1. The median patient age was 61 years (range 47–74). Nine patients (11.3%) had other malignant tumours, and 59 patients (90.7%) were men. ECOG performance status was 0–1 in 59 patients and 2 in 6 patients. The most common tumour histology was squamous (90.7%). Of all patients, 34 (52.3%) were newly diagnosed, and 31 (47.6%) had postoperative regional lymph node metastasis and had not received prior chemotherapy and radiotherapy. Among all newly diagnosed patients, 2/34 (5.8%) patients were stage II, 10/34 (29.4%) were stage III and 22/34 (64.7%) were stage IV. Two had tumours in the cervical region, 4 in the upper thoracic region, 16 in the middle thoracic region, 10 in the lower thoracic region and 2 in multiple synchronous primary sites.

**Table 1:** Clinical characteristics of 65 patients analysed in this study

Clinical parameter	*n* (%)
Age (years)	
Mean	61
Range	47–74
Gender	
Male	59 (90.7)
Female	6 (9.2)
Histology	
Squamous	59 (90.7)
Adenocarcinoma	4 (6.1)
Poorly differentiated carcinoma	2 (3.0)
Location of newly diagnosed tumor	36 (52.3)
Cervical	2 (3.0)
Upper thoracic	4 (6.1)
Middle thoracic	16 (24.6)
Lower thoracic	10 (15.3)
Synchronous multiple primary	2 (3.0)
ECOG^a^ score	
0–1	59 (90.7)
2	6 (9.2)
Stage (UICC^b^ 6th edition)	
Newly diagnosed	34 (52.3)
II	2 (3.0)
III	10 (15.3)
IV	22 (33.8)
Postoperative LNM^c^	31 (47.6)
Planned dose of radiation	
61.2 Gy	45 (69.2)
50.4 Gy	20 (30.7)

^a^Eastern Cooperative Oncology Group

^b^Union for International Cancer Control

^c^Lymph node metastasis

### Feasibility

Among all patients, 45 had a planned radiation dose of 61.2 Gy and 42 (93.3%) completed radiotherapy treatment. Of the 20 patients with a planned radiation dose of 50.4 Gy, 19 (95.0%) patients finished the complete radiotherapy regimen. A total of 61 patients (93.8%) completed treatment based on our definition for completing the radiotherapy regimen. Four (6.1%) patients did not finish radiotherapy because of heart disease (1), grade III oesophagitis (1), esophageal fistula (1) or esophageal bleeding (1).

A ≥5-cycle dose of concurrent chemotherapy with paclitaxel and carboplatin during radiotherapy was given to 63 patients (96.9%), and a full dose of 7 cycles of chemotherapy was given to 44 patients (67.6%). Two patients (3.0%) did not finish the 5-cycle dose of concurrent chemotherapy because of grade III thrombocytopenia (1) or ventricular arrhythmia (1).

### OS and PFS rates

**Fig. 3:**
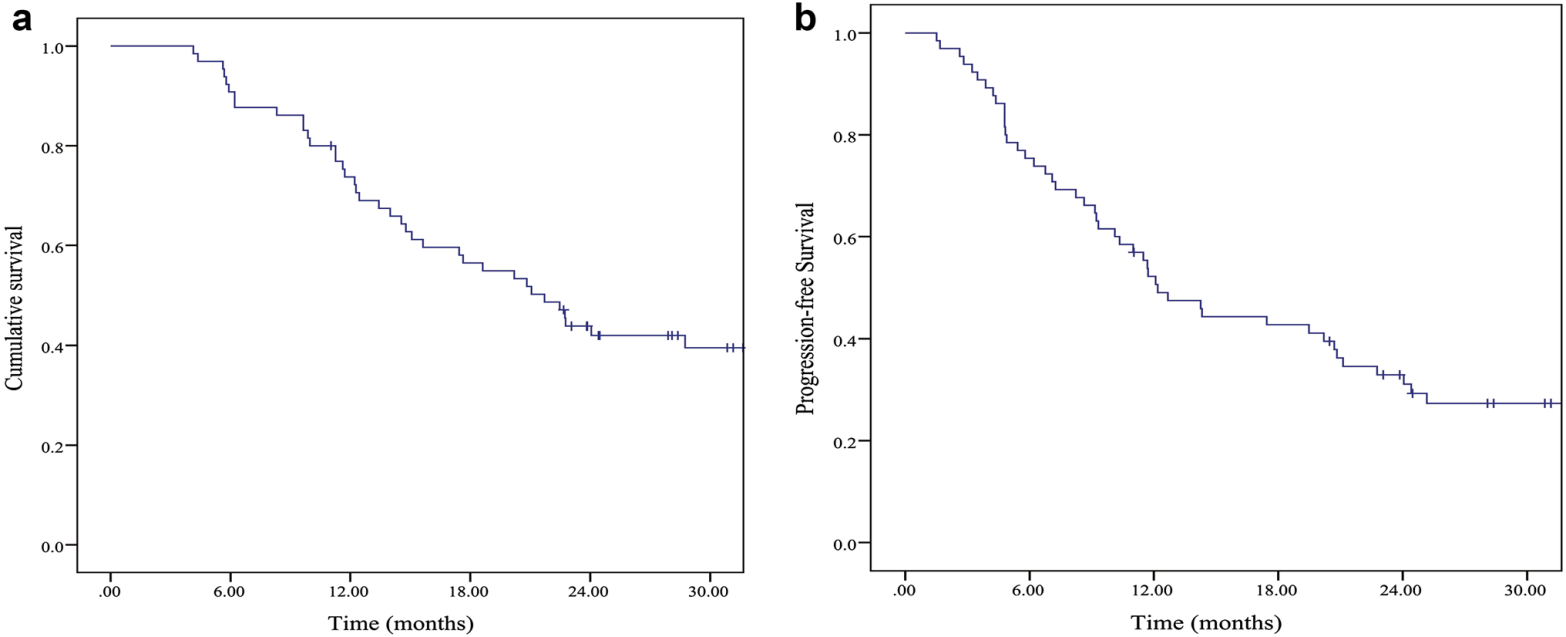
Overall survival (**a**) and progression-free survival (**b**) for 65 patients; the 1- and 2-year overall survival rates were 73.7% and 42.0%, respectively. 1- and 2-year progression-free survival rates were 50.6% and 31.1%, respectively

The median follow-up time was 26.3 months. At the time of our analyses, 27 patients were alive and 19 patients had no evidence of disease progression. The 1- and 2-year OS rates were 73.7 and 42.0%, respectively. The 1- and 2-year PFS rates were 50.6 and 31.1%, respectively. The median OS time was 21.7 months (95% confidence interval [CI] 16.7–26.6) and the median PFS time was 12.1 months (95% CI 9.0–15.3). Kaplan–Meier curves for the OS and PFS time are shown in Fig. 3. The 1- and 2-year local control rates were 75.4 and 68.9%, respectively. The 2-year OS was 32.1% for the newly diagnosed group and 53.3% for the postoperative recurrence group, with median survival of 18.6 and 28.7 months, respectively (*P* = 0.155). The median OS was 24.0 months for the radiation dose of 61.2 Gy and 14.7 months for the dose of 50.4 Gy (*P* = 0.204). The 2-year OS rate was 42.8% for the squamous carcinoma and 50.0% for adenocarcinoma (*P* = 0.274). A total of 46 patients (70.7%) had treatment failure. Thirteen patients had only locoregional failure, 28 patients had only distant metastasis, 4 patients had concurrent locoregional/distant failure, and 1 patient failed therapy as a result of heart disease. The treatment failure patterns of the 46 patients are presented in Table 2. At the time of analysis, 32 patients had distant metastases. The sites of metastases comprised the lungs (23 patients, 35.3%), distant lymph nodes (19 patients, 29.2%), bones (11 patients, 16.9%), liver (10 patients, 15.3%), pleura (4 patients, 6.1%), skin (2 patients, 3.0%), spleen (2 patients, 3.0%) and brain (2 patients, 3.0%). Among the locoregional failures in 16 patients, 11 patients had local regional recurrence and 5 patients had persistent local/regional disease.

**Table 2:** Location of disease at first treatment failure

	*n*	%
Local/regional only	13	20.0
Distant only	28	43.0
Local, regional and distant	4	6.1
Other	1	1.5
Total	46	70.7

### Toxicity

The toxicity and tolerability of the therapy were evaluated in all patients. The toxicities of chemoradiotherapy (graded according to NCI-CTC 4.0) are outlined in Table 3. Eighteen patients (27.6%) experienced grade 3 or higher severe events in this study. The most frequent acute adverse events for grades 3 and 4 were neutropenia, thrombocytopenia and oesophagitis. Grades 3 and 4 neutropenia were observed in 12 (18.4%) and 1 (6.1%) patients, respectively. Grades 3 and 4 thrombocytopenia, oesophagitis, anaemia, fatigue and pneumonitis occurred in 3/65 (4.6%), 3/65 (4.6%), 1/53 (1.5%), 1/65 (1.5%) and 1/65 (1.5%) cases, respectively. The other types of adverse reactions related to treatment were mild and included hypoalbuminaemia, peripheral sensory neuropathy and pericardial effusion. No allergic reaction was recorded in any of the patients. The grade 3 radiation dermatitis rate was 1.5%. There were 3 patients who experienced oesophageal fistula.

**Table 3:** Treatment toxicity

Grade	1	2	3	4
*N* = 65	*n* (%)	*n* (%)	*n* (%)	*n* (%)
Neutropenia	19 (29.2)	20 (30.7)	12 (18.4)	4 (6.1)
Thrombocytopenia	19 (29.2)	10 (15.3)	3 (4.6)	0
Anaemia	27 (41.5)	15 (23.0)	1 (1.5)	0
Vomiting	7 (10.7)	4 (6.1)	1 (1.5)	0
Fever	1 (1.5)	2 (3.0)	0	0
Fatigue	9 (13.8)	6 (9.2)	1 (1.5)	0
Alopecia	10 (15.3)	6 (9.2)	0	0
Hypoalbuminaemia	13 (20.0)	3 (4.6)	0	0
ALT^a^	3 (4.6)	0	0	0
Radiation dermatitis	8 (12.3)	8 (12.3)	1 (1.5)	0
Oesophagitis	19 (29.2)	19 (29.2)	3 (4.6)	0
Hypocalcaemia	8 (12.3)	1 (1.5)	0	0
Hypomagnesaemia	2 (3.0)	1 (1.5)	0	0
Hyponatraemia	8 (12.3)	0	0	0
Hypophosphataemia	4 (6.1)	0	1 (1.5)	0
Constipation	2 (3.0)	6 (9.2)	0	0
Diarrhoea	1 (1.5)	2 (3.0)	0	0
Peripheral sensory neuropathy	2 (3.0)	12 (18.4)	0	0
Pneumonitis	5 (7.6)	7 (10.7)	1 (1.5)	0
Hypopotassaemia	3 (4.6)	0	0	0
Hoarseness	7 (10.7)	2 (3.0)	0	0
Ventricular arrhythmia	3 (4.6)	0	0	0
Pericardial effusion	4 (6.1)	0	0	0

Toxicity was graded according to the National Cancer Institute Common Toxicity Criteria, version 4.0

^a^Alanine aminotransferase increased

## Discussion

In our phase II study, the regimen of weekly paclitaxel and carboplatin with concurrent radiotherapy was a promising approach to treating advanced oesophageal cancer in terms of the OS, local control rate and low rate of side effects. Toxicities of grade 3 or higher were 27.6% (18/65). The common adverse reactions were bone marrow suppression, oesophagitis and fatigue. The 1- and 2-year OS rates were 73.7 and 42.0%, respectively. The median OS time was 21.7 months. The 1- and 2-year PFS rates were 50.6% and 31.1%, respectively.

Compared to cisplatin and 5-fluorouracil (PF) with concurrent radiotherapy in RTOG 85-01 and RTOG 94-05, the regimen of paclitaxel and carboplatin produced less toxicity and increased OS [3, 12, 13]. In the RTOG 85-01 study, the median survival duration was 14.1 months with a 5-year survival rate of 26% in the chemoradiotherapy treatment group [3, 12]. Additionally, grade 3–4 adverse reactions occurred in 46% of the patients. In the RTOG94-05 study, patients were treated with radiotherapy doses of 64.8 and 50.4 Gy with PF, and the median survival times were 13.0 and 18.1 months, respectively [13]. The incidences of grade 3–5 acute toxicity in the high-dose and low-dose groups were 76 and 71%, respectively. In our study, the median OS was 21.7 months, and all patients had unresectable or recurrent oesophageal cancer, including 22/34 (64.7%) previously untreated patients diagnosed in stage IV.

Paclitaxel and cisplatin (TP) regimens are the most commonly reported paclitaxel-based regimens. Compared with cisplatin, the administration of carboplatin is convenient and does not require planned hydration and considerably shortens the duration of outpatient attendance. Adelstein et al. reported the use of TP in a phase II study [14]. The median survival was 15 months for patients with stage T3, N1 or M1 (nodal) oesophageal cancer, lower than that observed in our study. Neutropenia (95%) and nausea (95%) were the most frequent grade III/IV toxicities, and 16 patients (40%) experienced neutropenic fever, which must be addressed. This toxicity is significant and was higher than that in our study. Lawrence et al. reported a phase II study of neoadjuvant chemoradiotherapy with weekly dose TP compared with irinotecan plus cisplatin for resectable oesophageal adenocarcinoma [15]. The median OS survival time in the TP group was 21 months; however, acute toxicities grade 3–4 occurred in 65.2%, with relatively higher haematological toxicity. In the RTOG 0113 study, one group was treated with paclitaxel plus cisplatin and a radiotherapy dose of 50.4 Gy, and grades 3 and 4 adverse reactions occurred in 43 and 40% of the patients, respectively [16]. The TP regimen might have higher haematological toxicity, similar to the results of our previous phase II study of concurrent chemoradiotherapy for patients with inoperative oesophageal cancer [17]. In our study, the grade ≥3 haematological toxicity rate was 27.6%, lower than the results reported in a TP-based group study. The median OS was 21.7 months, which was comparable with the TP regimen studies reported.

The CROSS study showed that neoadjuvant chemoradiation combined with carboplatin and paclitaxel concurrent with radiation significantly prolonged OS, and did not increase surgery-related mortality compared with surgery alone [18]. Thus, preoperative chemoradiotherapy with TC regimen is considered to be a standard of care among patients with resectable oesophageal cancer. Meerten et al. reported a phase 2 study of definitive chemoradiotherapy with weekly dose paclitaxel (50 mg/m^2^) and carboplatin (AUC = 2) [19]. The median OS was 17 months. The grade 3 and 4 adverse events were neutropenia (16%), oesophagitis (12%), fatigue (8%) and pneumonitis (2%). Honing et al. retrospectively compared definitive chemoradiotherapy with TC to PF [20]. The median OS and PFS were 13.8 and 9.7 months, respectively. A higher percentage of patients with TC completed treatment compared with the PF group (82% vs 57%, *P* = 0.010). Van Ruler et al. retrospectively reviewed 66 patients treated with chemoradiation therapy with TC regimen [21]. The median OS was 13.1 months. 91 and 82% of patients, respectively, completed the full course of radiotherapy and 5 courses. In our study, the concurrent chemoradiotherapy completed was 90.7%, and the median OS was 21.7 months. Our results are comparable in terms of completed treatment and survival using the same chemoradiotherapy regimens.

Few studies have evaluated definitive chemoradiation therapy that included 3 cytotoxic drugs [22, 23]. Miyazaki et al. reported a phase II study of definitive chemoradiation therapy with docetaxel, cisplatin and 5-fluorouracil (DCF) in advanced oesophageal cancer [22]. The 2-year OS rate and median survival time were 52.9% and 24.7 months, respectively. However, the incidence of leukopenia and neutropenia was 91.9 and 75.7%, respectively. The KDOG 0501-P2 trial reported the use of DCF in a phase II study; the median survival was 29.0 months with a survival rate of 43.9% at 3 years [23]. Grade 3 or higher major toxicity consisted of leukopenia (71.4%), neutropenia (57.2%) and oesophagitis (28.6%). The clinical complete response (cCR) rate ranged from 54.1 to 52.4%. The DCF regimen appears to be highly active and to have relatively higher toxicity.

The SCOPE1 trial was a phase 2/3 study assessing the benefits of adding cetuximab to definitive chemoradiation therapy (capecitabine + cisplatin + radiation) to treat patients with localised oesophageal cancer [24]. The 2-year survival was 41.3% in the CRT plus cetuximab group and 56.0% in the CRT only group. The CRT plus cetuximab group also had shorter median OS (22.1 vs 25.4 months, respectively; *P* = 0.035) and more non-haematological grade 3 or 4 toxicities (79% vs 63%, *P* = 0.004). In our study, adverse events such as myelosuppression and oesophagitis were relatively mild.

Toxicity rates for TC in our study were comparable with the rates in the paclitaxel-based trial. The grade ≥3 haematological toxicity rates of TP with concurrent radiotherapy were 47–95% [14, 15, 17, 25]. In our study, 27.6% of patients experienced grade 3–4 hematological toxicities and 12.3% non-hematological toxicities, which was lower than that of the TP regimen. In our previous phase II study of paclitaxel and fluorouracil (TF) with concurrent radiotherapy, the occurrence of grade ≥3 or higher severe events was 32% and that of haematological events was 16.9% [26]. However, 8 patients experienced fever and 2 patients died from radiation pneumonitis in the TF regimen. Long-term continuously infused 5-FU might lead to higher incidence of oesophagitis and fever.

The limitations of our study are as follows. (1) The homogeneity of the patients in the group was poor; there were patients in phases II–III and IV, and there was postoperative recurrence. (2) The radiation dose was not uniform. Although the standard radiotherapy dose is 50.4 Gy in Europe and the United States, the radiotherapy dose for oesophageal cancer in China is often ≥60 Gy. Only normal tissue could not tolerate the use of 50.4 Gy in our study. Wang et al. reported that patients who received a radiation dose of ≥50 Gy had a better outcome than those who received <50 Gy [27]. Additionally, there was no statistical difference in the survival rates of patients with adenocarcinoma and squamous cell carcinoma, probably because the number of patients with adenocarcinoma was too small.

In conclusion, our study indicates that weekly infused paclitaxel and carboplatin, followed by 2-cycle consolidation chemotherapy, is a promising regimen with good tolerability for patients with oesophageal cancer. The limitation of our study is the small sample size. A randomised phase III trial (NCT02459457) with larger sample sizes is currently examining the efficacy of paclitaxel in combination with cisplatin, carboplatin or 5-fluorouracil concurrent with radiotherapy is on-going at our hospital.

## References

[1] Siegel RL, Miller KD, Jemal A (2017). Cancer statistics, 2017. CA Cancer J Clin.

[2] Pennathur A, Gibson MK, Jobe BA (2013). Oesophageal carcinoma. Lancet.

[3] Cooper JS, Guo MD, Herskovic A (1999). Chemoradiotherapy of locally advanced esophageal cancer: long-term follow-up of a prospective randomized trial (RTOG 85-01). Radiation Therapy Oncology Group. JAMA.

[4] al-Sarraf M, Martz K, Herskovic A (1997). Progress report of combined chemoradiotherapy versus radiotherapy alone in patients with esophageal cancer: an intergroup study. J Clin Oncol.

[5] Ajani JA, Ilson DH, Daugherty K (1994). Activity of taxol in patients with squamous cell carcinoma and adenocarcinoma of the esophagus. J Natl Cancer Inst.

[6] Choy H, Rodriguez FF, Koester S (1993). Investigation of taxol as a potential radiation sensitizer. Cancer.

[7] Choy H, Akerley W, Safran H (1998). Multiinstitutional phase II trial of paclitaxel, carboplatin, and concurrent radiation therapy for locally advanced non-small-cell lung cancer. J Clin Oncol.

[8] Choy H, Devore RF, Hande KR (2000). A phase II study of paclitaxel, carboplatin, and hyper fractionated radiation therapy for locally advanced inoperable non-small-cell lung cancer (a Vanderbilt Cancer Center Affiliate Network Study). Int J Radiat Oncol Bio Phys.

[9] Lau D, Leigh B, Gandara D (2001). Twice-weekly paclitaxel and weekly carboplatin with concurrent thoracic radiation followed by carboplatin/paclitaxel consolidation for stage III non-small-cell lung cancer: a California Cancer Consortium phase II trial. J Clin Oncol.

[10] van Meerten E, Muller K, Tilanus HW (2006). Neoadjuvant concurrent chemoradiation with weekly paclitaxel and carboplatin for patients with oesophageal cancer: a phase II study. Br J Cancer.

[11] van Hagen P, Hulshof MC, van Lanschot JJ (2012). Preoperative chemoradiotherapy for esophageal or junctional cancer. N Engl J Med.

[12] Herskovic A, Martz K, al-Sarraf M (1992). Combined chemotherapy and radiotherapy compared with radiotherapy alone in patients with cancer of the esophagus. N Engl J Med.

[13] Minsky BD, Pajak TF, Ginsberg RJ (2002). INT 0123 (Radiation Therapy Oncology Group 94-05) phase III trial of combined-modality therapy for esophageal cancer: high-dose versus standard-dose radiation therapy. J Clin Oncol.

[14] Adelstein DJ, Rice TW, Rybicki LA (2000). Does paclitaxel improve the chemoradiotherapy of locoregionally advanced esophageal cancer? A nonrandomized comparison with fluorouracil-based therapy. J Clin Oncol.

[15] Kleinberg LR, Catalano PJ, Forastiere AA (2016). Eastern Cooperative Oncology Group and American College Of Radiology Imaging Network randomized phase 2 trial of neoadjuvant preoperative paclitaxel/cisplatin/radiation therapy (RT) or irinotecan/cisplatin/RT in esophageal adenocarcinoma: long-term outcome and implications for trial design. Int J Radiat Oncol Biol Phys.

[16] Ajani JA, Winter K, Komaki R (2008). Phase II randomized trial of two nonoperative regimens of induction chemotherapy followed by chemoradiation in patients with localized carcinoma of the esophagus: RTOG 0113. J Clin Oncol.

[17] Tang H, Ma H, An SM (2016). A phase II study of concurrent chemoradiotherapy with paclitaxel and cisplatin for inoperable esophageal squamous cell carcinoma. Am J Clin Oncol.

[18] Shapiro J, van Lanschot JJB, Hulshof MCCM (2015). Neoadjuvant chemoradiotherapy plus surgery versus surgery alone for oesophageal or junctional cancer (CROSS): long-term results of a randomised controlled trial. Lancet Oncol.

[19] Marten EV, van Rij C, Tesselaar ME (2010). Definitive concurrent chemoradiation (CRT) with weekly paclitaxel and carboplatin for patients (pts) with irresectabe esophageal cancer: a phase II study. J Clin Oncol.

[20] Honing J, Smit JK, Muijs CT (2014). A comparison of carboplation and paclitaxel with cisplatinum and 5-fluorouracil in definitive chemoradiation in esophageal cancer patients. Ann Oncol.

[21] van Ruler MA, Peters FP, Slingerland M (2017). Clinical outcomes of definitive chemoradiotherapy using carboplatin and paclitaxel in esophageal cancer. Dis Esophagus.

[22] Miyazaki T, Sohda M, Tanaka N (2015). Phase I/II study of docetaxel, cisplatin, and 5-fluorouracil combination chemoradiotherapy in patients with advanced esophageal cancer. Cancer Chemother Pharmacol.

[23] Higuchi K, Komori S, Tanabe S (2014). Definitive chemoradiation therapy with docetaxel, cisplatin, and 5-fluorouracil (DCF-R) in advanced esophageal cancer: a phase 2 trial (KDOG 0501-P2). Int J Radiat Oncol Biol Phys.

[24] Crosby T, Hurt CN, Falk S (2013). Chemoradiotherapy with or without cetuximab in patients with oesophageal cancer (SCOPE1): a multicentre, phase 2/3 randomised trial. Lancet Oncol.

[25] Ilson DH, Forastiere A, Arquette M (2000). A phase II trial of paclitaxel and cisplatin in patients with advanced carcinoma of the esophagus. Cancer J.

[26] Xia Y, Li YH, Chen Y (2017). A phase II study of concurrent chemoradiotherapy combined with a weekly paclitaxel and 5-fluorouracil regimen to treat patients with advanced esophageal carcinoma. Radiat Oncol.

[27] Wang S, Liao Z, Chen Y (2006). Esophageal cancer located at the neck and upper thorax treated with concurrent chemoradiation: a single-institution experience. J Thorac Oncol.

